# Maternal mental health in Africa during the COVID-19 pandemic: a neglected global health issue

**DOI:** 10.4178/epih.e2021078

**Published:** 2021-10-06

**Authors:** Kobi V. Ajayi, Elizabeth Wachira, Obasanjo Afolabi Bolarinwa, Beulah D. Suleman

**Affiliations:** 1 Education, Direction, Empowerment, and Nurturing (EDEN) Foundation, Abuja, Nigeria; 2 Department of Health and Kinesiology, Texas A&M University, College Station, TX, USA; 3 Laboratory of Community Systems and Sciences, Texas A&M University, College Station, TX, USA; 4 Department of Health and Human Performance, Texas A&M University, Commerce, TX, USA; 5 Department of Public Health Medicine, School of Nursing and Public Health, University of KwaZulu-Natal, Durban, South Africa; 6 Obaxlove Consult, Lagos, Nigeria

**Keywords:** Coronavirus, Postpartum period, Pregnancy, Mental health, Africa

## Abstract

The coronavirus disease 2019 (COVID-19) pandemic has profoundly impacted mental health and well-being around the globe. Public health measures to control the virus’s rapid spread, such as physical distancing, social isolation, lockdown, restricted movements, and quarantine, caused fear and panic in the general population. Although pandemic-related stressors have been reported, changes that occur during the perinatal period compounded by those made to obstetric care guidelines may put pregnant and postpartum mothers at an increased risk of poor mental health. While an abundance of research has examined the impact of the pandemic on maternal mental health in developed nations such as Europe and America, very few studies have done so in the African continent. Considering that Africa has prominently weak health systems, poor mental health policies and infrastructure, high poverty rates, and unreliable maternal care, the pandemic is expected to have dire consequences on maternal mental health in the region. As such, multipronged mental health interventions and strategies that consider the heterogeneity within and between African regions must be developed. Doing so will close existing and widening global health disparities to achieve the United Nations Sustainable Development Goals by 2030.

## INTRODUCTION

Research has shown that the coronavirus disease 2019 (COVID-19) pandemic has profoundly impacted mental health and well-being around the globe. Measures to mitigate the rapid spread of the virus, such as physical distancing, social isolation, lockdown, restricted movements, and quarantine, elicited fears and worries among the general population [1,2]. Additionally, concerns for personal safety, misinformation and hoaxes about COVID-19 and the vaccine, and adverse socioeconomic consequences exacerbated mental health problems [1]. Although poor mental health outcomes due to the pandemic abound, pregnant and postpartum women are at a higher risk of mental health issues than the general population [3].

While this article focuses on maternal mental health during the pandemic in Africa, it is germane to highlight the epidemiological evidence supporting the adverse psychological sequelae of the pandemic on various population subgroups. A review of mental health consequences amid the pandemic found that patients with COVID-19, pre-existing psychiatric disorders, and healthcare workers had higher psychiatric symptoms, including posttraumatic stress symptoms, depression, and a decrease in overall psychological well-being [2]. This positive association between poor mental health and epidemics or public health emergencies is attributable to public health prevention strategies, and this pattern has been reported in previous epidemics [4]. An umbrella review of mental health outcomes of infection control measures such as quarantine and other prevention measures found that depression, anxiety disorders, mood disorders, posttraumatic stress symptoms, panic, stigmatization, and sleep disorders, are common among individuals who observe social distancing [4]. Although several factors (e.g., a poor social network or lower educational status) have exacerbated mental health burdens amid the pandemic [2], being woman is an already well-known risk factor for mental health conditions [5]. As a result, there has been no shortage of research and public health efforts addressing the disproportionate mental health consequences during pregnancy and postpartum [3,6].

Epidemiological data indicate that women are twice as likely as men to experience mental health problems, and hormonal changes during the perinatal period further increase women’s susceptibility to mood and anxiety disorders [5]. Thus, it is not surprising that since the pandemic, pregnant and postpartum women have reported higher proportions of mental health problems than those who are not pregnant or in the postpartum period [3]. The reasons for these disparate observations are not far-fetched. For example, pregnant women with COVID-19 have a higher risk of severe illness than non-pregnant women with the virus. In addition, COVID-19 increases the risk of adverse birth outcomes (e.g., preterm birth) [7]. These negative obstetric consequences of the pandemic increased stress, worries, and anxiety and are unique to pregnancy [8]. In addition to these concerns, postpartum women must also cope with caregiving burdens. Indeed, this period is undeniably challenging for those with multiple children as they navigate parenting and familial responsibilities while also worrying about the COVID-19 pandemic. Moreover, disruptions to maternal care and perinatal mental health services have contributed to poor maternal mental health [8].

Additionally, significant alterations have been made to obstetric care protocols, including but not limited to visitation restrictions, changes to birth plans, wearing of face masks during delivery, and cancellation or modifications to antenatal visits to mitigate the cross-transmission of the virus to mothers and their newborns [9]. Simultaneously, psychosocial needs unique to the perinatal period have been adversely impacted in the COVID-19 response. These findings indicate that, while necessary, infection control and public health intervention measures have unintended consequences that may increase mental health burdens among this already vulnerable population, particularly those with pre-existing trauma and in resource-constrained settings, such as Africa.

While research has shown that the COVID-19 has severe adverse repercussions on maternal mental health, these studies are limited to developing nations [3]. Hence, there is limited knowledge of the magnitude of the pandemic’s effects on maternal mental health and well-being in developing countries. Therefore, this paper discusses the implications of the COVID-19 pandemic on maternal mental health in Africa.

## GLOBAL EVIDENCE OF MATERNAL MENTAL HEALTH IN COVID-19

Globally, consistent findings have been reported concerning the impact of the pandemic on maternal mental health [3,9-13]. A review of 8 articles from Italy, Canada, China, Turkey, and Greece comprising 7,750 pregnant or postpartum women with symptoms of depression and anxiety before and after the COVID-19 pandemic found a high and significant overall pooled State-Trait Anxiety Inventory (STAI) score [3]. A cross-sectional study using a web-based survey among 1,873 pregnant women in China used the validated Chinese Perceived Stress Scale (cut-off, > 25), Self-Rating Anxiety Scale (cut-off, > 50), and Chinese Edinburgh Depression Scale (cut-off, > 10) to assess stress, anxiety, and depression, respectively. The prevalence of stress, anxiety, and depression was 89.1% (95% confidence interval [CI], 87.6 to 90.4), 18.1% (95% CI, 16.4 to 19.9), and 45.9% (95% CI, 43.6 to 48.1), respectively [10].

Elevated levels of perinatal depression and anxiety have also been reported in longitudinal studies. In Argentina, 204 women (102 pregnant women and 102 non-pregnant women) were accessed for symptoms of depression and anxiety using the Spanish adaptation of the Beck Depression Inventory-II, the Spanish version of the STAI, and the Spanish adaptation of the Positive and Negative Affect Schedule at 3 different time points (2, 14, and 47 days after the start of the lockdown) [11]. This study found statistically significant higher scores for depression and anxiety at time point 3 (p < 0.01) among pregnant women than among non-pregnant women. Moreover, there were significant differences in reduction in positive affect at time point 3 (p < 0.05) between pregnant and non-pregnant women. Similarly, in the United States, researchers used ecological momentary assessments to measure stress levels among pregnant and postpartum women at 4 different time points (pre-pandemic, during the time of the first few reported cases in the United States, during the stay-at-home orders, and post-lockdown) [12]. This study demonstrated that mean stress levels increased significantly between the second (0.26 points, p< 0.001) and fourth time points (0.2 points, p= 0.002) compared to the pre-pandemic phase.

In the same vein, qualitative studies suggest that women are anxious, worried, and stressed across several thematic categories. For example, pregnant women in Turkey reported receiving insufficient or conflicting information from their healthcare providers, not understanding the severity of the COVID-19 virus, and having to delay care deliberately or go without care because of fear of contracting the virus [13]. Beyond these sources of negative emotional feelings, pregnant and new mothers from 8 countries (the United States, Canada, United Kingdom, Switzerland, Australia, Spain, Jamaica, and the Philippines) who shared their birth stories on YouTube during the pandemic noted that they felt alone and had to self-advocate for appropriate care because they had no support during labor and delivery [9]. This study also revealed that mothers who tested positive for COVID-19 received delayed care because their nurses were afraid of contracting the infection. At the same time, mothers with infants in the neonatal intensive care unit struggled with the stringent visitation policies, which made bonding with their infants challenging [9].

Nonetheless, despite these findings, evidence demonstrates that there may be positive aspects of the pandemic among pregnant women and postpartum mothers. For example, some mothers who gave birth during the COVID-19 pandemic, when hospital policy allowed only 1 partner to be present during labor and delivery, described their birthing experience as peaceful [9].

## MATERNAL MENTAL HEALTH IN AFRICA PRE-COVID-19

Despite the high rates of mood and anxiety disorders among mothers reported across Africa pre-COVID-19, there is a conspicuous lack of maternal mental health research in Africa amid the pandemic. According to the World Health Organization, 15.6% of pregnant women and 19.8% of those with recent childbirth in developing nations experience a mental health disorder, compared to 10% and 13% globally [14]. Moreover, another study found that the prevalence of prenatal and postnatal depression and anxiety in Africa was 11.3% and 18.3% and 14.8% and 14.0%, respectively [15], a higher rate than those of 6.5% to 12.9% found in developed nations [16]. Although it is plausible that differences in the prevalence between regions are attributable to variations in psychological measurement tools, the majority of studies utilized well established, reliable, and validated psychological measurement tools, including but not limited to the Edinburgh Postnatal Depression Scale, Mini International Neuropsychiatric Interview, General Health Questionnaire, and Schedule for Affective Disorders and Schizophrenia. Thus, other contextual factors such as healthcare systems may account for the discrepancies, albeit to a limited extent. It appears then that there are underlying factors aggravating the risk of symptoms of depression and anxiety among perinatal women in Africa, which puts them at a higher risk of poor mental health and quality of life than those in developed nations.

Accordingly, the high rates of depressive and anxiety symptoms in Africa are likely to have increased because of the unintended consequences of infection control measures. For instance, lockdown measures have greatly impacted individuals employed in the informal sectors, among whom women are disproportionately represented, increased gender-based violence, caused losses of earning or employment, and led to disrespectful maternity care due to the COVID-19 [17]. Moreover, the pandemic has limited access to reproductive healthcare, family planning, and preventive health services, with the greatest burden in the African region. Indeed, these conditions expose women to a greater risk of poor mental health. A recent study during the COVID-19 shutdown found a disproportionately elevated risk of depression and anxiety among women with a history of intimate partner violence (IPV) and sexual violence than among men (24.0 vs. 6.4% and 16.1 vs. 3.5%, respectively) [18]. In that study, the likelihood of mild (adjusted odds ratio [aOR], 1.64; 95% CI, 1.05 to 2.55) and moderate (aOR, 2.60; 95% CI, 1.53 to 4.41) mental health symptoms were significantly associated with a history of IPV [18]. This demonstrates that the pandemic has had disparate consequences on women as compared to men.

Furthermore, there is a stark shortage of mental health research in Africa, necessitating heightened attention. For example, of the 637 articles referencing Africa within 5 years since the inception of the *Lancet Global Health* journal, only 21 were related to mental health [19]. However, this is not surprising considering that low-and-middle-income countries have historically received a smaller fraction of global health resources for mental health [17]. Another pressing concern is the low mental health workforce and service utilization in the region. Africa has 1.4 mental health workers per 100,000 people compared to the global average of 9.0 per 100,000 people. The utilization of mental health treatment in Africa is also below the global average (14 per 100,000 vs. 1,051 per 100,000) [19].

Additionally, given the high rates of poverty and socioeconomic inequalities, weak health systems, poor obstetric care protocols and maternal health services, and the region’s growing population [20], it is reasonable to conclude that pregnant and postpartum women in Africa will experience a magnified impact of the pandemic and its full scale that has yet to be understood. Insights from the 2014-2016 Ebola outbreak, although deadlier and less contagious than COVID-19 which led to long-term mental health consequences must be also be considered [21]. Considering the devastating outcomes of maternal mental health disorders on mothers, children, families, and society [3], it is an injustice and a disservice that the global community has focused mainly on maternal mental health in developed countries.

## MATERNAL MENTAL HEALTH IN AFRICA DURING THE COVID-19 PANDEMIC

There is evidence that some regions in Africa have instituted some form of mental health and psychological guidelines in response to the pandemic [22]. Although promising, these responses are inadequate to meet the region’s mental health needs. Conversely, alarmingly high rates of suicide in Malawi, Nigeria, and South Africa have been linked to COVID-19 [23-25]. More so, the literature shows there is poor mental health among the general population during COVID-19 in Africa [1,26]. Surprisingly, investigations and guidelines pertinent to maternal mental health disorders have largely been neglected. It appears then that the psychological well-being of pregnant and postpartum women in Africa has been excluded from consideration in the COVID-19 response despite abundant evidence about their vulnerability. To test this hypothesis, we conducted electronic database and gray literature searches using the “OR” and “AND” Boolean combinations iteratively to search for relevant articles published between January 2020 and August 2021. The keywords used included but were not limited to “covid-19,” “coronavirus,” “2019-ncov,” “pregnancy,” “pregnant,” “prenatal,” “antenatal,” “perinatal,” “maternal,” “postnatal,” “postpartum,” “anxiety,” “depression,” “mental health,” “subsaharan Africa,” “western Africa, “eastern Africa,” “southern Africa,” and “northern Africa” to identify existing literature to map out the impact of the pandemic on maternal mental health in Africa. Unsurprisingly, however, we found only 4 articles from Ethiopia, Ghana, and Nigeria [27-30], supporting the position that COVID-19-related mental health policies and programs in Africa are scarce.

Kassaw & Pandey [27] in Ethiopia conducted a hospital-based analysis of 178 women seeking perinatal services using the General Anxiety Disorder-7 tool. In their study, the prevalence of general anxiety disorder was 32.2%, a higher rate than was found in China [10]. Another study in Ethiopia investigated psychological distress and self-efficacy among 384 pregnant women receiving follow-up antenatal care using the Impact of Event Scale Revised (IES-R), Hospital Anxiety and Depression Scale, Generalized Self-Efficacy Scale, and Fear of COVID-19 scale [28]. The findings of this study indicated severe psychological distress (IES-R scale mean score, 45.1± 17.4). The author also found a significant positive association between symptoms of anxiety and depression and fear of COVID-19. However, further analysis revealed that self-efficacy was a protective factor against psychological distress and had a negative association with fear of COVID-19 [28]. In Ghana, Moyer et al. [29] assessed anxiety and healthcare-seeking behavior among 71 pregnant women using an online survey distributed via social networks. Although the study found that 66.2% and 71.0% of women were anxious about being pregnant or giving birth during COVID-19, the assessment tools used were non-validated. Nevertheless, an overwhelming 87.0% of women feared getting sick with COVID-19, 65.2% feared the stigma associated with getting sick, and 63.8% reported worries about discrimination if infected with COVID-19 [29].

Researchers in Nigeria measured symptoms of depression, anxiety, and stress symptoms among 456 pregnant women attending antenatal care using the Depression Anxiety Stress Scale-21 during the lockdown [30]. In this study, 23.0% of women had severe stress, while 16.7% had extremely severe stress. Furthermore, 7.2% and 6.4% of women, respectively, reported severe depression and extremely severe depression, and severe anxiety and extremely severe anxiety were recorded in 3.3% and 7.7% of women, respectively. The evidence showed consistent relationships between psychological risk factors and mental health during the pandemic. For example, higher levels of education, parity, and being in the third trimester predicted poor mental health in Nigeria and Ethiopia [27,28,30]. Similarly, urban residence was associated with a higher psychological burden [27,30]. These patterns mirror those found in developed countries, urgently necessitating investigations of the true effect of the pandemic on maternal mental health in Africa.

## IMPLICATIONS FOR MATERNAL MENTAL HEALTH POLICY AND PRACTICE IN AFRICA

Given that maternal mental health issues are complex, multipronged mental health interventions and strategies are needed to mitigate the impact of the pandemic on pregnant and new mothers: (1) Effective policies focused on healthcare financing, strengthening existing mental health systems, and collaborative research and partnerships between institutions are needed. These can help ensure proper surveillance of the maternal mental health situation and conduct research to inform service provision protocols and policy-making. Moreover, having a maternal mental healthcare plan where regular screenings, referral mechanisms, and treatment services are offered as part of prenatal and postnatal care assures that service is systematic and can assist with continued surveillance. In addition, primary care providers and pediatricians regularly interact with mothers; therefore, the integration of mental healthcare into this context is possible, especially when guided by a maternal mental health care plan [8]; (2) This service integration and continued surveillance require current service providers to be trained on screening and counseling services, supervision, and staff monitoring. Additionally, since most African countries rely heavily on community health workers and volunteers to deliver services, they too should be trained. For instance, training on case detection and referrals, counseling, psychosocial screenings, administering psychological first-aid, and understanding the need for referral to professionals are achievable goals [17]; (3) Culturally adapted psychological screening tools specific to African women during and beyond the pandemic should be developed. This approach may address health beliefs, practices, and societal and cultural needs unique to Africa while also attenuating barriers to care. In the same vein, a culturally competent and diverse behavioral workforce might also facilitate respectful and responsive maternal mental healthcare; (4) Access to mental health services remains an issue in most African countries. In addition to capacity building, telepsychiatry approaches to provide mental health services have been implemented and are on the rise. For example, phone calls, video calls, and toll-free numbers have effectively reached more people with counseling and mental health advice, especially in remote locations [22]. Albeit effective, innovative approaches are still needed to cater to those in vulnerable areas where individuals with low digital literacy and limited or no access to the Internet and power live. Employing mass media for communication to provide easy-to-understand information and resources for self-help measures have been found effective in resource-constrained settings and must also be utilized [17,22]. Additionally, spousal and community support should be encouraged; and (5) Finally, there is a pressing need for robust population-based quantitative, ecological, and rigorous qualitative research to fully understand the pandemic’s magnitude and the views of pregnant and postpartum women to inform maternal mental health policy, research, and programs in Africa during and beyond the pandemic.

## CONCLUSION

In Africa, maternal mental health must be prioritized to achieve the United Nations Sustainable Development Goals by 2030 and close the widening disparities caused by the COVID-19 pandemic. Understanding the heterogeneity between and within the African region is key for employing context-specific mental health initiatives to mitigate the adverse maternal health consequences of the pandemic on pregnant and new mothers. Furthermore, as countries gradually ease lockdowns, despite ongoing mutations of the coronavirus and the emergence of new variants, the African region must rethink its approach to maternal mental health not only to mitigate the effects of the COVID-19 pandemic, but also to provide a blueprint for future public health emergencies.

### Ethics statement

Not applicable since this manuscript does not involve any experimentation.
